# Uncovering the dynamics of extracellular vesicle microRNA trafficking in multiple sclerosis: delivery, diversion and retention

**DOI:** 10.20517/evcna.2026.11

**Published:** 2026-05-28

**Authors:** Gemma C. Stevenson, Manisha S. Patil, Georges E.R. Grau

**Affiliations:** ^1^Vascular Immunology Unit, Infection, Immunity & Inflammation Theme, School of Medical Sciences, Faculty of Medicine and Health, University of Sydney, Sydney 2050, Australia.; ^2^The University of Sydney Nano Institute, Sydney Nanoscience Hub, Sydney 2050, Australia.

**Keywords:** Extracellular vesicles, miRNA, multiple sclerosis, exosomes, microvesicles, immunopathology, cytokines

## Abstract

Extracellular vesicles (EVs) are phospholipid-bound structures that carry a distinct repertoire of short, non-coding microRNAs (miRNAs). EV miRNA content differs across various diseases, including multiple sclerosis (MS). These observations may indicate a functional role for EV miRNAs in disease pathology. However, the role of EVs in miRNA trafficking remains a subject of debate. This review proposes a theoretical framework to understand the dynamics of EV miRNA trafficking in MS. Three modes of EV miRNA trafficking are presented: (i) delivery of miRNA to target cells; (ii) diversion of miRNA away from its target; or (iii) retention of the miRNA in the parent cell. This model accounts for the heterogeneity and functional diversity of EVs and may facilitate the identification of physicochemically distinct subpopulations with selective roles in miRNA transfer.

## INTRODUCTION

Extracellular vesicles (EVs) are phospholipid-bound structures with key physiological and pathological functions. EVs facilitate intercellular communication by transporting biomolecules between proximal and distal cells. This cargo includes a repertoire of microRNAs (miRNAs) which alter gene expression when delivered to a recipient cell^[1-3]^.

Changes in EV miRNA levels are reported across many neuroinflammatory conditions, including multiple sclerosis (MS)^[3-17]^. MS is a chronic, autoimmune disease characterised by inflammation, demyelination and axonal degeneration^[18]^. Immune dysregulation is a prominent feature of MS. Patients exhibit a breakdown of peripheral immune tolerance, including effector cell resistance to T regulatory (Treg) cell activity, and infiltration of autoreactive cells into the central nervous system (CNS). Nevertheless, the mechanisms underlying these pathological changes in immune function remain unresolved.

EVs isolated from MS patients are enriched in miRNAs that target pathways associated with inflammation, immune regulation, oxidative stress, and CNS function^[13]^. The ability of EVs to transport these miRNAs across the blood-brain barrier (BBB) indicates a role for EV miRNAs in the pathological mechanisms underpinning MS^[19,20]^. Distinct patterns of EV miRNA expression are also reported across MS subtypes^[4,6,17]^ and treatment regimens^[5,7]^ as well as during relapse^[13]^. These findings provide strong evidence supporting a functional role for EV-associated miRNAs in MS.

Although EV-derived miRNAs have the potential to influence MS pathology by altering gene expression, the functional role of EVs in miRNA transport remains the subject of debate. On one side, there are questions as to whether EVs can deliver enough miRNA to induce functional changes in gene expression^[21]^. However, these concerns are countered by a growing body of evidence demonstrating the functional delivery of miRNAs to recipient cells^[2,22-24]^.

In this review, we address this debate by proposing a novel conceptual framework for understanding the roles of EVs in miRNA trafficking. We propose that EVs may deliver miRNAs to target cells, divert miRNAs away from the parent cell, or facilitate the retention of miRNAs in the parent cell. These subpopulations of EVs may exhibit physicochemical differences that underpin their functional specialisation. We then apply this functional framework to the observed patterns of EV miRNA and target gene expression in MS. In line with the MISEV recommendations, we use the generic term ‘EV’ to encompass all phospholipid membrane-bound vesicle subpopulations reported in the literature^[25]^.

## DELIVERY, DIVERSION AND RETENTION

### Delivery

Here, EV-mediated miRNA delivery is defined as the transfer of miRNAs from a parent to a recipient cell, resulting in measurable changes in target gene expression. The capacity of EVs to facilitate intercellular miRNA delivery was first reported by Valadi *et al*., who described this cargo as “exosomal shuttle RNA”^[26]^. Their study demonstrated that specific messenger RNA (mRNA) and miRNA species are selectively packaged into EVs and transported between mast cells to alter gene expression. Numerous studies have since confirmed these findings across immunological^[22]^, neuronal^[23]^ and viral^[27]^ contexts.

Despite this body of evidence, there are concerns that the concentration of miRNA within EVs is insufficient to induce functionally relevant changes in gene expression. Chevillet *et al*. analysed the miRNA content of EVs isolated from human plasma, seminal fluid and cell culture supernatants^[28]^. They reported an average EV miRNA concentration of one miRNA per 121 EVs, with concentrations ranging from one miRNA per nine EVs in seminal fluid, to one copy per 47,162 EVs in healthy donor plasma. These findings were confirmed by Albanese *et al*. who determined that EV miRNAs were a minor proportion of the total extracellular miRNA pool^[21]^.

Nevertheless, these findings may be interpreted in two ways: either all EVs contain very low miRNA concentrations (low occupancy, low miRNA concentration), or a rare subpopulation of EVs contains very high miRNA concentrations (low occupancy, high miRNA concentration). The second interpretation supports a model in which a rare class of EVs, with specific physicochemical properties, could deliver miRNAs and alter gene expression in the recipient cell.

#### Mechanisms of EV miRNA delivery

EV miRNA delivery requires a coordinated sequence of events. miRNAs must first be loaded into the EV and trafficked to the cell membrane for release. The EV must then evade peripheral clearance and selectively engage with its target cell. The EV may then fuse with the plasma membrane or be endocytosed and escape the endolysosomal pathway. These constraints on EV miRNA delivery support an interpretation where functionally distinct EVs bypass extracellular and intracellular clearance mechanisms to transport miRNAs between cells.

The first step in EV miRNA delivery is miRNA loading. As these mechanisms have been reviewed elsewhere^[29,30]^, we will provide a summary of these processes and how they are influenced by environmental cues. Sorting motifs play a key role in miRNA loading into EVs. Sorting motifs are recognised by RNA-binding proteins for selective miRNA packaging. Numerous RNA-binding proteins are involved in EV miRNA loading, including members of the heterogeneous nuclear ribonucleoprotein (hnRNP) family^[31-33]^, as well as the ribonucleoprotein, fragile X messenger ribonucleoprotein 1 (FMR1)^[34]^.

Extrinsic signals can modulate RNA-binding protein activity and influence EV miRNA loading. For example, insulin signalling triggers phosphorylation of the hnRNPA1^[33]^. Phosphorylated hnRNPA1 preferentially bound to a subset of miRNAs and loaded them into EVs. Similarly, inflammation has also been shown to affect EV miRNA packaging. Inflammasome activation increases cytoplasmic levels of the cleaved Rab-interacting lysosomal protein (RILP)^[34]^. Cleaved RILP enhanced FMR1 binding to specific miRNA motifs and increased FMR1 association with the endosomal sorting complex required for transport (ESCRT) machinery. This resulted in a selective enrichment of pro-inflammatory miRNAs, including miR-155.

Once released from the parent cell, the ‘delivery’ EV must evade peripheral clearance and destruction. The expression of surface proteins such as CD47 may allow EVs to avoid these clearance mechanisms. CD47 interacts with signal regulatory protein α on macrophages to inhibit phagocytosis^[35]^. EVs expressing CD47 are less likely to be phagocytosed and are more likely to successfully deliver their RNA cargo to recipient cells^[36]^. EV lipid composition has also been shown to affect phagocytosis. Phosphatidylserine is exposed on the outer leaflet of the plasma membrane during apoptosis to promote phagocytic clearance. EVs with surface-exposed phosphatidylserine are preferentially phagocytosed by macrophages^[37]^. These findings suggest that lower phosphatidylserine concentrations are favourable for EV persistence in circulation.

Having evaded peripheral clearance, the EV must selectively engage with its recipient cell. Numerous surface ligands are suggested to promote the selective uptake of EVs by their target cells. These interactions can be cell specific or generalised across cell types. For example, Wang *et al*. identified a neuron-specific EV uptake mechanism involving Notch receptor-ligand interactions^[38]^. Alternatively, fibronectin is a widely expressed protein that has been shown to interact with heparan sulphate to facilitate EV uptake^[39]^. Interestingly, fibronectin has also been found to engage with integrin β1 and integrin α4, enabling EV endocytosis and functional RNA delivery^[40]^.

The final barrier to EV miRNA delivery is degradation within the endolysosomal pathway. The majority of endocytosed EVs are shuttled through the endolysosomal system where they are destroyed by lysosomal enzymes. Nevertheless, a proportion of EVs can escape the early endosome and successfully deliver their miRNA cargo into the cytoplasm^[41]^. The mechanisms underlying endosome escape include endosomal fusion, permeabilisation or rupture of the endosomal membrane^[42]^.

### Diversion

EVs have primarily been considered for their role in cargo delivery. However, equal consideration should be given to EVs whose cargo is packaged but not delivered to a recipient cell. Here, EV-mediated miRNA diversion refers to the release of miRNA-enriched EVs which are trafficked away from the parent cell for peripheral clearance.

Diversion may be a particularly important mechanism under cellular stress or pathological conditions. Intracellular RNA and miRNA balance can determine whether miRNAs are retained or diverted via EVs^[43]^. Artificial overexpression of a miRNA or target mRNA results in miRNA colocalisation at multivesicular bodies (MVB) and P bodies. This colocalisation increases miRNA packaging into EVs, allowing the excess miRNA to be diverted away from the parent cell.

The potential for EVs to support cell homeostasis under pathological conditions has also been reported for other non-RNA cargo. For example, cells employ a secretory autophagy mechanism in response to lysosomal dysfunction and impaired autophagosome maturation. This mechanism targets autophagic intermediates for release within EVs to compensate for cellular dysfunction^[44]^. Similarly, EVs preserve cell homeostasis by diverting fragmented chromosomal DNA away from the parent cell^[45]^. The diversion of fragmented DNA in EVs prevents reactive oxygen species-dependent DNA damage responses which could lead to apoptosis.

Nevertheless, the fate of diverted EVs is less clear. It is plausible that this subpopulation of EVs is physicochemically distinct from those capable of delivering miRNA to recipient cells. The functional specialisation of these EVs may be due to an absence of inhibitory signals to prevent clearance, or a specific composition optimised for miRNA disposal, clearance or redistribution.

### Retention

The low levels of miRNA within EVs have previously been seen as evidence that EVs cannot transport miRNAs between cells^[21]^. However, these observations may not represent a failure of delivery. miRNAs may be selectively retained within their parent cells and actively withdrawn from the EV pool. The coordinated withdrawal of miRNAs from EVs could have equally significant physiological or pathological effects as miRNA delivery or diversion.

Retention of cellular miRNAs may be essential in maintaining intracellular RNA homeostasis and regulating downstream gene expression. For example, the artificial depletion of intercellular miRNAs has been shown to reduce their packaging into EVs^[43]^. Whilst this may be a passive process of maintaining homeostasis, there is evidence of selective miRNA retention in response to remote ischaemic preconditioning^[46]^. The withdrawal of miRNAs from the EV repertoire was suggested to relieve suppression of downstream stress-adaptive pathways. In this way, miRNA retention was demonstrated to act as a subtractive regulatory mechanism to protect against future ischaemic injury.

Selective mechanisms of cellular miRNA retention have also been reported. The miRNA-binding protein Poly-rC Binding Protein 2 (PCBP2) has been shown to regulate miRNA retention by inhibiting Synaptotagmin-Binding, Cytoplasmic RNA-Interacting Protein (SYNCRIP)-dependent EV miRNA loading^[47]^. Interestingly, the protein, hnRNA2B1 is also suggested to play a role in cellular miRNA retention by preventing hsa-miR-503 loading into EVs^[48]^. It is notable that miR-503 does not express the same signal motif reported to facilitate hnRNA2B1-mediated miRNA loading in previous studies^[32]^. This suggests that hnRNA2B1 has two independent mechanisms of regulating EV miRNA packaging: one to facilitate miRNA loading via sequence motifs, and one to retain cellular miRNAs.

## EV MIRNA TRANSPORT ACROSS THE BBB

**Figure 1:**
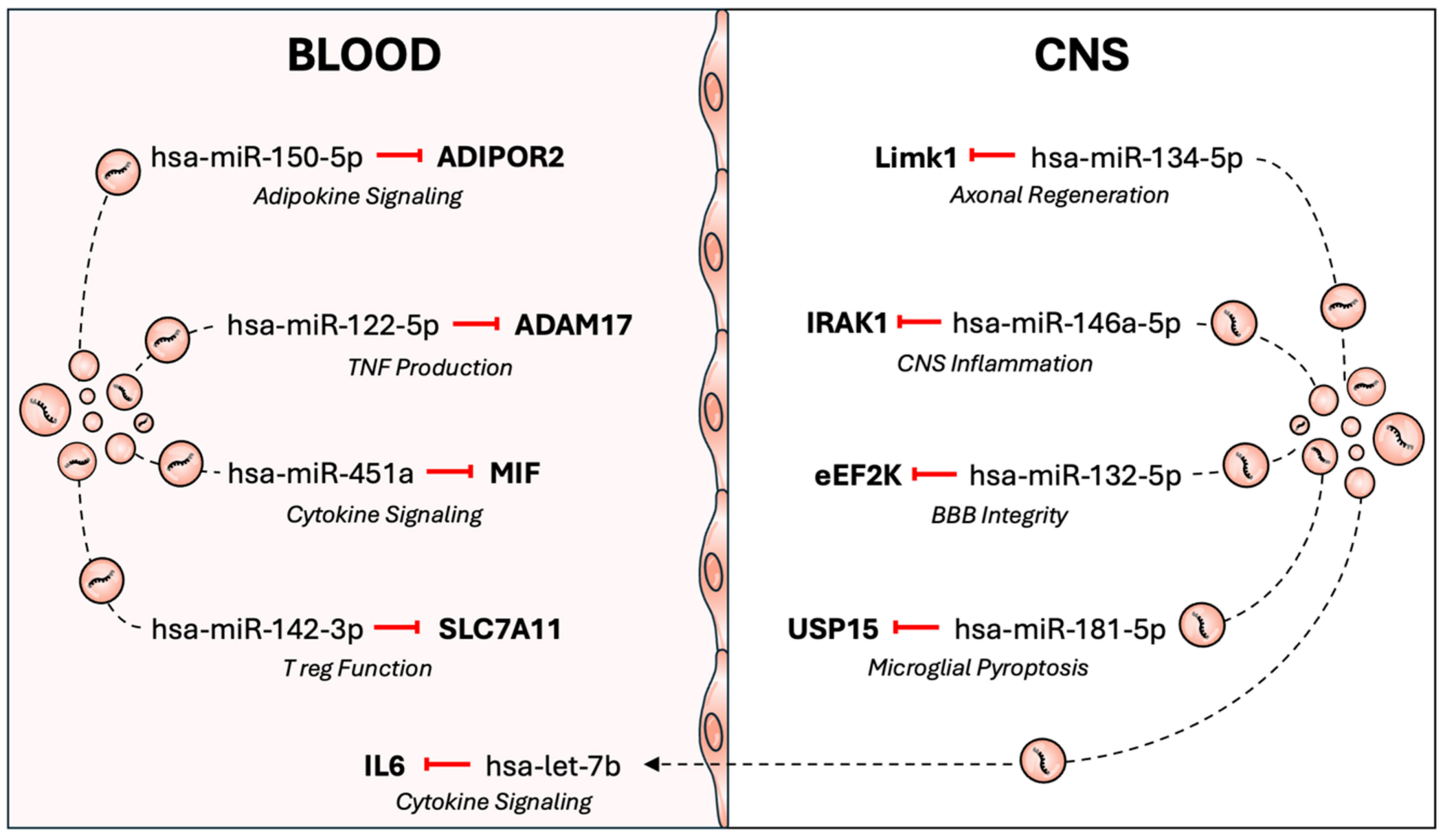
EV miRNAs may contribute to disease activity in multiple sclerosis. EVs can transport miRNAs across the blood-brain barrier, facilitating a bidirectional crosstalk between the periphery and the central nervous system. EVs may also transport miRNAs within the central nervous system or blood compartments. The dysregulated loading of miRNAs into EVs has the potential to influence MS pathology by altering immunological, CNS and viral gene expression. ADAM17: ADAM metallopeptidase domain 17; ADIPOR2: adiponectin receptor 2; BBB: blood-brain barrier; CNS: central nervous system; eEF2K: eukaryotic elongation factor 2 kinase; EV: extracellular vesicle; IL6: interleukin 6; IRAK1: interleukin-1 receptor-associated kinase 1; Limk1: LIM domain kinase 1; MIF: macrophage migration inhibitory factor; miRNA: microRNA; MS: multiple sclerosis; SLC7A11: solute carrier family 7 member 11; TNF: tumor necrosis factor; USP15: ubiquitin specific peptidase 15; T reg: regulatory T cell.

The BBB is a selective interface between the peripheral circulation and CNS. The potential for EVs to transport miRNAs across the BBB is of significant relevance to MS [Figure 1]. The presence of peripheral cell-associated miRNAs in cerebro-spinal fluid (CSF) EVs and CNS-associated miRNAs in plasma EVs provides observational evidence of bidirectional EV exchange between the blood and CNS compartments.

The two primary mechanisms involved in EV transport across the BBB are the paracellular and transcellular pathways^[49]^. Paracellular transport involves the passage of EVs through the intercellular junctions between endothelial cells. However, this mechanism is poorly characterised for EVs and is unlikely to be the primary pathway involved. Alternatively, transcellular transport involves EV endocytosis, passage through the cell and release of the intact vesicle at the opposite surface.

The initial endocytosis of an EV can occur through caveolin-dependent or caveolin-independent transcytosis mechanisms. Treating induced pluripotent stem cell-derived endothelial cells with filipin, an inhibitor of caveolin-dependent endocytosis, significantly reduced the passage of erythrocyte EVs across a transwell model^[50]^. These findings supported a caveolin-dependent mechanism of EV trafficking across the BBB. However, in another study, filipin treatment had no effect on the passage of tumour-derived EVs across a similar transwell system of human brain endothelial cells^[51]^. These EVs were instead shown to be taken up by clathrin-mediated endocytosis then sorted into rab11^+^ endosomes and released at the basolateral membrane through a vesicle-associated membrane protein 3 (VAMP3)/synaptosome-associated protein of 23 kDa (Snap23)/syntaxin4-dependent process. These findings may indicate that the mechanisms of endocytosis differ by EV origin, model system or extrinsic cues. Nevertheless, they support the notion that an active mechanism of transcellular transport facilitates the transport of EVs between the blood and CNS.

Inflammation is a characteristic feature of MS pathophysiology and may enhance the probability of EV miRNA delivery across the BBB. Stimulating endothelial cells with the pro-inflammatory cytokine, tumour necrosis factor (TNF), has been demonstrated to enhance EV passage across an *in vitro* transwell system^[52]^. This effect was attenuated by inhibitors of both clathrin-dependent and caveolin-dependent endocytosis. Such findings illustrated that pro-inflammatory signals could increase EV trafficking via a transcellular, not paracellular route. A similar effect on EV transcytosis was observed after treatment with lipopolysaccharide; however, the mechanisms of transcytosis were not further explored^[20]^.

## EV MIRNA DELIVERY, DIVERSION AND RETENTION IN MS

Distinct patterns of EV miRNA expression have been reported across MS disease subtypes, therapies and disease activity states^[3-17]^. These EVs carry a dysregulated repertoire of miRNAs involved in neuro- and peripheral inflammation, T cell function, BBB maintenance and remyelination.

The unique pathological environment of MS may drive changes in EV miRNA packaging, composition, and downstream processes of delivery, diversion, or retention. Oxidative stress can produce characteristic EV miRNA profiles, likely by altering miRNA or target mRNA expression in the parent cell^[53]^. Hypoxia, which is often observed in MS lesions^[54]^ can also regulate the activity of ESCRT-associated proteins and divert MVBs away from the lysosomal pathway. Similarly, inflammation can alter RNA-binding protein activity and EV miRNA packaging^[34]^. Nevertheless, whether dysregulated EV miRNA expression is a cause or consequence of MS-associated pathologies remains unclear.

### EV miRNA delivery in MS

The potential for EVs to regulate gene expression via miRNA delivery makes them of significant interest in understanding the immunological and CNS pathologies underpinning MS [Table 1].

**Table 1:** EV miRNA and MS target gene expression dynamics aligning with a delivery mode of EV miRNA trafficking

**EV miRNA**	**Source**	**Expression pattern**	**Cohort**	**MS-related target gene**
hsa-miR-146a-5p	CSF	Upregulated	RRMS *vs*. control^[8]^	IRAK-1
	Serum	Downregulated	RRMS IFN-β *vs*. treatment naïve^[7]^	
hsa-miR-134-5p	Serum	Upregulated	Fingolimod non-responsive RRMS *vs*. responsive^[5]^	Limk1
hsa-miR-150-5p	Serum	Upregulated	RRMS *vs*. control^[9]^	ADIPOR2
	CSF	Upregulated	RRMS *vs*. control^[9]^	
	Serum (IB4^+^ EVs)	Upregulated	RRMS cognitively impaired *vs*. cognitively preserved^[10]^	
	Serum	Downregulated	RRMS fingolimod *vs*. treatment naïve^[5]^	
hsa-let-7i	Plasma	Upregulated	MS *vs*. Control^[14]^	IGF1R TGFBR1

Limk1: LIM domain kinase 1; IRAK-1: interleukin-1-receptor associated kinase 1; IFN-β: interferon beta; ADIPOR2: adiponectin receptor 2; IGF1R: insulin-like growth factor 1 receptor; TGFBR1: transforming growth factor beta receptor 1; RRMS: relapsing-remitting multiple sclerosis; CIS: clinically isolated syndrome; S/PPMS: secondary/primary progressive multiple sclerosis.

Immune dysregulation is a key feature of MS pathology. EV-mediated delivery of hsa-miR-146a-5p and hsa-let-7i may contribute to such dysregulation by targeting key inflammatory and regulatory pathways. Both hsa-miR-146a^[8]^ and hsa-let-7i^[14]^ are upregulated in EVs isolated from MS patients. Therapeutic downregulation of EV hsa-miR-146a has also been reported in relapsing-remitting multiple sclerosis (RRMS) patients after interferon-β (IFN-β) therapy^[7]^.

hsa-miR-146a-5p has been shown to downregulate interleukin-1 receptor-associated kinase-1 (IRAK-1) expression^[55]^. The miRNA-mediated repression of IRAK-1 was demonstrated to induce a compensatory upregulation of IRAK-2 in stressed astroglial cells, potentially driving CNS inflammation^[55]^. The potential for EVs to cross the BBB and deliver hsa-miR-146a-5p to astroglial cells within the CNS is of considerable relevance in MS. CNS inflammation is a prominent feature of MS which contributes to oxidative stress, BBB dysfunction and neuronal death^[56]^. Notably, single nucleotide polymorphisms in both hsa-miR-146a and IRAK-1 are associated with increased MS risk^[57]^. Whilst IRAK expression has not been assessed in MS patients, the upregulation of this miRNA in MS patient EVs may contribute to the CNS inflammation observed in MS.

MS patients also exhibit a pathological imbalance between Treg and T effector cell function^[58]^. This imbalance may be exacerbated by the upregulation of EV-associated hsa-let-7i. EV hsa-let-7i levels are inversely correlated with Treg cell frequency in MS patients^[14]^. EVs have been demonstrated to deliver hsa-let-7i to CD4+ T cells. Once delivered, hsa-let-7i repressed insulin-like growth factor 1 receptor (IGF1R) and transforming growth factor beta receptor 1 (TGFBR1) protein expression and inhibited Treg induction and proliferation. The increased delivery of hsa-let-7i may hinder peripheral tolerance mechanisms and contribute to the dysregulated immune environment observed in MS.

EV miRNA delivery may also contribute to CNS dysfunction in MS. hsa-miR-134-5p expression has been associated with pathological brain activity in other CNS conditions, including epilepsy^[59]^. The pathological effects of hsa-miR-134-5p expression were attributed to its repression of LIM domain kinase 1 (Limk1). Limk1 is a signalling protein involved in maintaining structural plasticity and facilitating axonal regeneration after neural injury^[60]^. This is supported by reports that inhibiting hsa-miR-134-5p expression could restore neuronal plasticity in Alzheimer’s disease^[61]^. Given these findings, the upregulation of hsa-miR-134-5p in MS patient EVs may increase its delivery to the CNS, where it inhibits neuronal regeneration via Limk1.

Finally, EVs may deliver miRNAs involved in systemic inflammatory signalling. For example, hsa-miR-150-5p has been demonstrated to repress adiponectin receptor 2 (ADIPOR2) expression^[62]^. ADIPOR2 is the cellular receptor for the adipokine, adiponectin^[63]^. Adiponectin enhances Treg cell function and reduces oxidative stress. However, adiponectin expression is positively correlated with increased MS disease activity and worse disease prognosis^[64,65]^. Given that adiponectin should be protective in MS patients, EV delivery of hsa-miR-150-5p could downregulate ADIPOR2 expression in recipient cells and prevent it from exerting its anti-inflammatory effect.

The dynamics of EV miRNA and target gene expression presented in this section support a mode of EV-mediated miRNA delivery. There is significant potential for this delivery mechanism to be exploited for therapeutic use or targeted for disease intervention. For example, blocking EV-mediated delivery of these miRNAs could prevent downstream pathological effects resulting from target gene repression. Alternatively, understanding how EVs package and target miRNAs for delivery could enable the production of miRNA-enriched EVs designed to counteract pathological imbalances in disease states.

### EV miRNA diversion in MS

Alongside their role in miRNA delivery, a subset of EVs may divert miRNAs away from both parent and recipient cells. It is plausible that a shift from miRNA delivery to diversion could drive changes in gene expression that contribute to disease activity in MS [Table 2].

**Table 2:** EV miRNA and MS target gene expression dynamics aligning with a diversion mode of EV miRNA trafficking

**EV miRNA**	**Source**	**Expression pattern**	**Cohort**	**Target genes**
hsa-miR-451a	Serum	Upregulated	RRMS *vs*. control^[6]^	MIF
	Serum	Upregulated	RRMS *vs*. control and CIS^[4]^	
	Serum	Downregulated	IFN-β responsive RRMS *vs*. non-responsive^[7]^	
hsa-miR-374a-5p	Serum	Upregulated	RRMS *vs*. control^[9]^	FOXO1
	CSF	Upregulated	RRMS *vs*. control^[9]^	
	Serum	Upregulated	RRMS *vs*. S/PPMS^[6]^	

MIF: Macrophage migration inhibitory factor; FOXO1: forkhead box protein O1; RRMS: relapsing-remitting multiple sclerosis; CIS: clinically isolated syndrome; S/PPMS: secondary/primary progressive multiple sclerosis.

hsa-miR-451a represses the expression of macrophage migration inhibitory factor (MIF)^[66-68]^. MIF is a pleiotropic cytokine with roles in cell proliferation, immune function, and the induction of caspase-independent cell death^[69]^. Increased MIF expression has been observed in active MS lesions^[70,71]^ as well as in the serum and CSF of RRMS patients^[72]^. However, this increase in target gene expression is inconsistent with the upregulation of hsa-miR-451a in MS patient EVs. The decoupling of target gene and miRNA expression indicates that EV-associated hsa-miR-451a is not repressing MIF expression in MS patients. This pattern is consistent with the diversion model. The increased packaging and disposal of EV-associated hsa-miR-451a could reduce its availability to repress MIF expression. This may allow for continued MIF expression and hence contribute to the pro-inflammatory environment characteristic of MS.

hsa-miR-374a-5p is another miRNA where upregulated EV levels do not correspond with target gene activity in MS. hsa-miR-374a-5p has been demonstrated to repress Forkhead box protein O1 (FOXO1) expression^[73,74]^. FOXO1 is a transcription factor involved in T helper cell proliferation and inducible Treg cell development^[75]^. Notably, inhibiting FOXO1 reduces T cell encephalitogenicity in MS patient T cells^[75]^. Furthermore, FOXO1 downregulation is necessary for Treg cells to maintain immune tolerance^[76]^. These findings suggest that FOXO1 signalling could contribute to immune imbalances in MS patients. Such imbalance in FOXO1 signalling could be driven by EV-associated diversion of hsa-miR-374a-5p.

The corresponding upregulation of EV miRNAs and target genes support a mode of EV-mediated miRNA diversion. miRNA diversion could occur passively if the parent cell was overexpressing an miRNA, or actively through selective packaging and disposal. Passive miRNA diversion may be favourable under physiological conditions to maintain mRNA:miRNA homeostasis. However, it could become pathological if recipient cells overexpress the target mRNA while the parent cell does not. Alternatively, dysfunction of selective miRNA packaging processes could result in miRNAs to be diverted, rather than delivered to a recipient cell.

### EV miRNA retention in MS

Retention is the final mode of miRNA trafficking described in this framework. This mechanism may occur if an miRNA is under expressed in the parent cell relative to its target mRNA or if an miRNA is selectively excluded from the EV repertoire. The retention of these miRNAs has the potential to alter intracellular regulatory balance, affecting immune or CNS functions relevant to MS [Table 3].

**Table 3:** EV miRNA and MS target gene expression dynamics aligning with a retention mode of EV miRNA trafficking

**EV miRNA**	**Source**	**Expression pattern**	**Cohort**	**Target genes**
hsa-miR-142-3p	Plasma	Upregulated	RRMS *vs*. control^[9]^	SLC7A11
	CSF	Downregulated	RRMS *vs*. control^[9]^	
	Plasma (Treg EVs)	Downregulated	RRMS *vs*. control^[3]^	
	Plasma	Downregulated	RRMS IFN-β *vs*. treatment naïve^[7]^	
hsa-let-7b	Plasma (IB4^+^ EVs)	Downregulated	RRMS cognitively impaired *vs*. cognitively preserved^[10]^	IL-6
	Serum	Downregulated	RRMS IFN-β *vs*. treatment naïve^[7]^	
hsa-miR-122-5p	Serum	Downregulated	RRMS (active *vs*. stable)^[12]^	ADAM17 (TACE)
	Serum	Upregulated	Fingolimod non-responsive RRMS *vs*. responsive^[5]^	
	Serum	Downregulated	IFN-β responsive RRMS *vs*. non-responsive^[7]^	
hsa-miR-223-3p	Serum	Upregulated	RRMS *vs*. S/PPMS^[6]^	RBPJ
	Plasma	Upregulated	SPMS *vs*. Stable RRMS^[17]^	
	Serum	Downregulated	RRMS *vs*. CIS and control^[4]^	
	Serum	Downregulated	RRMS IFN-β *vs*. treatment naïve^[7]^	
hsa-miR-132-5p	Serum	Downregulated	RRMS *vs*. control^[9]^	eEF2K
	CSF	Downregulated	RRMS *vs*. control^[9]^	
hsa-miR-181a-5p	Plasma (IB4^+^ EVs)	Downregulated	RRMS cognitively impaired *vs*. cognitively preserved^[10]^	USP15

SLC7A11: Solute carrier family 7 member 11; IL6: interleukin 6; ADAM17: a disintegrin and metalloproteinase 17; RBPJ: recombination signal binding protein for immunoglobulin kappa J region; eEF2K: eukaryotic elongation factor 2 kinase; USP15: ubiquitin-specific protease 15; RRMS: relapsing-remitting multiple sclerosis; CIS: clinically isolated syndrome; S/PPMS: secondary/primary progressive multiple sclerosis.

MS patients often exhibit imbalances in T effector cell and Treg function. The EV-associated miRNA hsa-miR-142-3p is suggested to contribute to this imbalance through a pathological retention mechanism^[3]^. Normally, Treg-derived EVs traffic hsa-miR-142-3p to conventional T cells. hsa-miR-142-3p suppresses T cell proliferation and activity by downregulating the cystine/glutamate antiporter, Solute Carrier Family 7 Member 11 (SLC7A11). However, in MS patients, the impaired packaging of hsa-miR-142-3p into EVs leads to its accumulation in the parent Treg. This suppresses Treg proliferation and thus, drives an imbalance between T effector and Treg cell activity. Nevertheless, there are contrasting reports of increased hsa-miR-142-3p in plasma EVs from RRMS patients^[9]^ and of its therapeutic downregulation in plasma EVs from RRMS patients after IFN-β therapy^[7]^. A plausible explanation for this divergent pattern is the distinct subpopulations of EVs studied. The dysfunctional retention of hsa-miR-142-3p by Treg cells may represent one component of a complex network of EV-associated miRNA trafficking.

The cellular retention of other miRNAs may also contribute to cytokine overexpression in MS. For example, hsa-miR-122-5p regulates the expression of a disintegrin and metalloproteinase 17 (ADAM17), also known as TNF-α-converting enzyme (TACE)^[77]^. ADAM17 is a cytokine-processing proteinase involved in TNF production^[78]^. This proteinase is highly expressed in active MS lesions, as well as in active microglia and parenchymal astrocytes of MS patients^[79]^. The reduction in EV hsa-miR-122-5p levels in MS patient EVs during active disease^[12]^ supports a model where cellular retention of hsa-miR-122-5p contributes to ADAM17 overexpression. In contrast, the increase in EV hsa-miR-122-5p reported by Ebrahimkhani *et al*. may reflect shifts in miRNA delivery, diversion, and retention across different stages of MS pathology, or in different subpopulations of EVs^[5]^.

A similar pattern of cellular retention may explain the dynamic between EV-associated hsa-let-7b and interleukin-6 (IL-6) expression. hsa-let-7b has been demonstrated to repress IL-6 expression^[80]^. IL-6 is a pleiotropic cytokine that influences a range of antigen-specific and inflammatory immune responses^[81]^. The overexpression of IL-6 in MS models has been shown to increase the resistance of autoreactive T effector cells to Treg suppression and promote the expansion of pathogenic Th17 cells^[82,83]^. The potential for hsa-let-7b to affect IL-6 expression is particularly significant in the context of IB4^+^ myeloid cell-derived EVs^[10]^. Myeloid cells are primary producers of IL-6^[84]^. The cellular retention of hsa-let-7b could be an attempt to regulate IL-6 mRNA expression and restore mRNA:miRNA homeostasis. However, this could also initiate a positive feedback loop. The cellular retention of hsa-let-7b could prevent its delivery to recipient cells and thus facilitate IL-6 overexpression in the parent cell.

Beyond the potential roles in immune dysfunction, EV miRNA retention could also affect CNS function and repair in MS patients. EV hsa-miR-181a-5p has previously been observed to alleviate CNS injury in the experimental autoimmune encephalitis model^[11]^. This was demonstrated by Shi *et al*., who showed that mesenchymal stem cell-derived EVs enriched in hsa-miR-181a-5p were trafficked to and delivered to microglia^[11]^. Once delivered, hsa-miR-181a-5p regulated microglial pyroptosis by repressing ubiquitin specific peptidase 15 (USP15) and downstream signalling via the USP15/REL-associated protein A (Rel A)/NIMA-related kinase 7 (NEK7) axis. Given this, a reduction in EV hsa-miR-181a-5p levels could drive microglial pyroptosis and CNS inflammation by relieving repression of the USP15 signalling pathway. This mechanism could explain the downregulation of EV hsa-miR-181a-5p in the myeloid-derived EVs of cognitively impaired MS patients.

Similarly, the downregulation of EV-associated hsa-miR-132-5p could contribute to BBB dysfunction in MS. EV-associated hsa-miR-132 has been demonstrated to regulate brain vascular integrity by repressing eukaryotic elongation factor 2 kinase (eEF2K)^[85]^. eEF2K modulates downstream VE-cadherin expression by phosphorylating its target protein, eukaryotic elongation factor 2 (eEF2). The reduced levels of hsa-miR-132-5p in RRMS patient serum and CSF EVs could contribute to the BBB dysfunction observed in MS patients before clinical relapse and disease onset.

Finally, human EV-miRNAs may also target genes that encode proteins involved in Epstein-Barr virus (EBV) gene expression. hsa-miR-223-3p has been shown to repress the expression of recombination signal-binding protein for immunoglobulin kappa J region (RBPJ)^[86]^. RBPJ is a cofactor for Epstein-Barr virus nuclear antigen 2 (EBNA2). The EBNA2-RBPJ complex enables the transactivation of latent viral genes^[87]^ and upregulation of MS-associated genes involved in B cell activation^[88]^. The reduced levels of hsa-miR-223-3p in MS patient EVs could represent a cellular retention of this miRNA. This would prevent hsa-miR-223-3p from repressing RBPJ expression. The resulting increase in RBPJ would allow for greater EBNA2-RBPJ binding to MS gene loci, thereby driving both EBV-dependent transcriptional programs in infected cells and increased expression of MS-associated susceptibility genes^[88]^.

## EV-ASSOCIATED EBV MIRNAS IN MS

Viruses have evolved mechanisms of hijacking host EV loading pathways to facilitate nucleic acid trafficking^[89]^. EBV is a herpesvirus with a strong association with MS. EBV encodes 44 miRNAs from 25 pre-miRNA precursors^[90]^. EBV has been shown to exploit host cell EV packaging pathways to deliver viral miRNA to host cells^[27]^. These findings were supported by the enrichment of MS patient EVs with EBV-miRNAs that have complete or partial matches to human EXOmotifs^[91]^. Given this, the presence of EBV miRNAs in MS patient EVs is of considerable interest in understanding how EBV may contribute to MS pathology.

EBV miRNAs have key roles in viral replication and persistence. These miRNAs can exert their activity by mimicking host miRNAs and modulating host gene expression. ebv-miR-BART9-3p (BART: BamHI A rightward transcript) is a prominent example of this. It has been shown to mimic the activity of hsa-miR-141 by repressing Forkhead box protein O3a (FOXO3a) expression^[92]^. Importantly, FOXO3a repression is necessary for EBV to initiate a productive lytic cycle. It is plausible that EBV could hijack host EV miRNA packaging pathways to amplify FOXO3a repression and transition into the lytic cycle. This would be consistent with the elevated levels of ebv-miR-BART9-3p in EVs isolated from the CSF of RRMS patients^[8]^. While the role of EBV reactivation in MS remains incompletely understood, a shift from the lysogenic to lytic cycle could trigger an inflammatory reaction that contributes to MS pathophysiology^[93]^.

Viral miRNAs can also interact with viral mRNAs. Indeed, ebv-miR-BART10-3p has been shown to target the 3’ untranslated region of the anti-apoptotic transcript BamHI H-fragment rightward open reading frame 1 (BHRF1)^[94]^. Significantly elevated plasma EV ebv-miR-BART10-3p levels are reported in RRMS patients after 12 months of high efficacy anti-CD20, natalizumab or fingolimod treatment^[16]^. Among these groups, the highest levels of ebv-miR-bART10-3p were observed in the EVs of RRMS patients undergoing anti-CD20 therapy. However, this upregulation contrasts with recent reports describing reduced ebv-miR-BART10-3p expression in the buffy coat fragment of peripheral blood samples from RRMS patients^[95]^. In this study, patients receiving anti-CD20 monoclonal antibodies had significantly lower ebv-miR-BART10-3p levels compared to treatment-naive MS patients and those receiving IFN-β therapy. The decoupling between EV and cellular miRNA expression may reflect an active mechanism of EV miRNA packaging, intended to offload the viral miRNA and prevent apoptosis.

The complex dynamics through which EBV may co-opt host EV miRNA packaging pathways represent an important avenue for future investigation. Understanding how the delivery, diversion or retention of EBV miRNAs could affect MS disease activity is necessary to characterise the host-pathogen dynamic and reveal novel therapeutic targets for MS.

## LIMITATIONS AND FUTURE DIRECTIONS

In this review, we have presented a novel conceptual framework for interpreting the dynamics of EV miRNA and target gene expression in MS. This model reinforces the functional heterogeneity of EVs in miRNA trafficking and associates the patterns of EV miRNA packaging with both the state of the parent cell and the disease context.

Nevertheless, this conceptual model requires experimental validation. This will require an understanding of the miRNA expression patterns that influence EV packaging, the surface markers that affect EV fate, as well as the minimal quantity of EV-delivered miRNAs necessary to induce functional changes in gene expression.

Live tracing of EVs, coupled with luciferase reporter assays and polymerase chain reaction (PCR)-based target gene expression analysis, could reveal the dynamics underlying EV delivery and their contributions to disease-associated gene expression. This could be coupled with multiplexed single-EV profiling methods to characterise miRNA content and surface marker expression at the individual level^[96]^. These techniques could then be used to assess how shifts in the stoichiometric balance between miRNA and target mRNA expression influence EV miRNA content, physicochemical properties or function.

EV miRNAs also have significant potential as biomarkers for disease progression and treatment efficacy. However, there are inconsistent reports on the direction of EV miRNA dysregulation. This variation is likely due to differences in cohort, EV subpopulation, or methods used for EV isolation. These methods require standardisation before EVs can be used as biomarkers of disease activity.

A further challenge is posed by the overlap between miRNAs dysregulated in MS patient EVs and those dysregulated in other neurological diseases, such as Alzheimer’s disease^[97]^, cerebral malaria^[98-100]^ and viral inflammation^[4]^. To overcome this barrier, it may be necessary to assess the coordinated expression of multiple EV-associated miRNAs. The composite miRNA profile could provide a more representative, specific, and reliable biomarker for MS.

This conceptual model provides a valuable framework for characterising the dynamic roles of EVs in miRNA trafficking. Whether an EV delivers, diverts or facilitates cellular retention of miRNAs likely reflects the cellular and environmental context. Understanding these dynamics and uncovering ways to exploit these mechanisms for therapeutic benefit would be invaluable for advancing the EV field.

## CONCLUSIONS

The conceptual framework outlined in this review provides a structured model for interpreting the functional significance of dysregulated EV-associated miRNAs in the context of MS pathophysiology. The three modes of EV-associated miRNA trafficking are (i) delivering miRNA to target cells, (ii) diverting the miRNA away from its target, or (iii) retaining the miRNA in the parent cell. These dynamics are reflected both in the EV literature, where there are divergent reports on EV function in miRNA delivery, and in the relationship between dysregulated EV miRNAs and their target gene expression in MS.

Our model reinforces the dynamic nature of EVs as a platform for miRNA trafficking. Experimental validation of this framework may reveal functionally distinct EV subpopulations that could be exploited for therapeutic use. These observations reinforce a growing body of evidence supporting the use of EVs as a platform for understanding disease pathologies, developing novel therapeutic tools, and identifying minimally invasive biomarkers for CNS disorders.

## DECLARATIONS

### Copyright

© The Author(s) 2026.
